# *N*-(2,5-Dimethyl­phen­yl)-2-nitro­benzene­sulfonamide

**DOI:** 10.1107/S1600536812047630

**Published:** 2012-11-24

**Authors:** U. Chaithanya, Sabine Foro, B. Thimme Gowda

**Affiliations:** a Department of Chemistry, Mangalore University, Mangalagangotri 574 199, Mangalore, India; b Institute of Materials Science, Darmstadt University of Technology, Petersenstrasse 23, D-64287 Darmstadt, Germany

## Abstract

In the crystal structure of the title compound, C_14_H_14_N_2_O_4_S, the N—H bond is *syn* to the *ortho*-nitro group in the sulfonyl benzene ring and *anti* to the *ortho*- and *syn* to the *meta*-methyl groups in the aniline ring. The mol­ecule is twisted at the S—N bond with a torsion angle of 71.41 (18)°. The dihedral angle between the planes of the benzene rings is 51.07 (8)°. In the crystal, pairs of N—H⋯O_sulfonamide_ hydrogen bonds link the mol­ecules into inversion dimers.

## Related literature

For studies on the effects of substituents on the structures and other aspects of *N*-aryl­sulfonamides, see: Chaithanya *et al.* (2012 ▶); Gowda *et al.* (2002 ▶) and of *N*-chloro­aryl­sulfonamides, see: Gowda & Shetty (2004 ▶); Shetty & Gowda (2004 ▶).
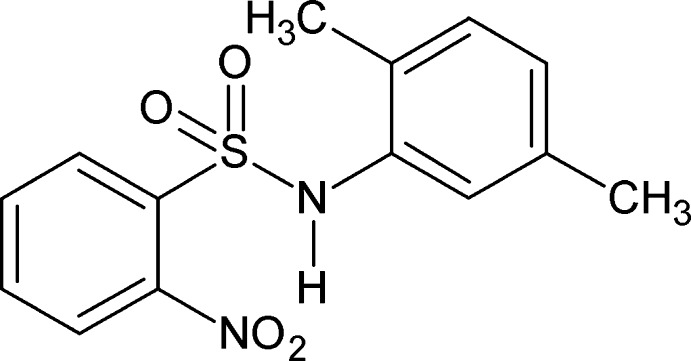


## Experimental

### 

#### Crystal data

C_14_H_14_N_2_O_4_S
*M* *_r_* = 306.33
Triclinic, 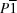

*a* = 8.1987 (7) Å
*b* = 9.6729 (9) Å
*c* = 9.9328 (9) Å
α = 84.386 (9)°
β = 72.096 (8)°
γ = 89.239 (9)°
*V* = 745.86 (12) Å^3^
*Z* = 2
Mo *K*α radiation
μ = 0.23 mm^−1^
*T* = 293 K
0.36 × 0.24 × 0.16 mm

#### Data collection

Oxford Diffraction Xcaliburdiffractometer with Sapphire CCD detector
Absorption correction: multi-scan (*CrysAlis RED*; Oxford Diffraction, 2009 ▶) *T*_min_ = 0.921, *T*_max_ = 0.964
4954 measured reflections
3027 independent reflections
2629 reflections with *I* > 2σ(*I*)
*R*_int_ = 0.011

#### Refinement

*R*[*F* ^2^ > 2σ(*F* ^2^)] = 0.042
*wR*(*F* ^2^) = 0.108
*S* = 1.14
3027 reflections
193 parameters
1 restraint
H atoms treated by a mixture of independent and constrained refinement
Δρ_max_ = 0.28 e Å^−3^
Δρ_min_ = −0.29 e Å^−3^

### 

Data collection: *CrysAlis CCD* (Oxford Diffraction, 2009 ▶); cell refinement: *CrysAlis CCD*; data reduction: *CrysAlis RED* (Oxford Diffraction, 2009 ▶); program(s) used to solve structure: *SHELXS97* (Sheldrick, 2008 ▶); program(s) used to refine structure: *SHELXL97* (Sheldrick, 2008 ▶); molecular graphics: *PLATON* (Spek, 2009 ▶); software used to prepare material for publication: *SHELXL97*.

## Supplementary Material

Crystal structure: contains datablock(s) I, global. DOI: 10.1107/S1600536812047630/rz5026sup1.cif

Structure factors: contains datablock(s) I. DOI: 10.1107/S1600536812047630/rz5026Isup2.hkl

Supplementary material file. DOI: 10.1107/S1600536812047630/rz5026Isup3.cml

Additional supplementary materials:  crystallographic information; 3D view; checkCIF report

## Figures and Tables

**Table 1:** Hydrogen-bond geometry (Å, °)

*D*—H⋯*A*	*D*—H	H⋯*A*	*D*⋯*A*	*D*—H⋯*A*
N1—H1*N*⋯O2^i^	0.82 (2)	2.27 (2)	3.023 (2)	152 (2)

Symmetry code: (i) 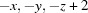.

**Fig. 1.**
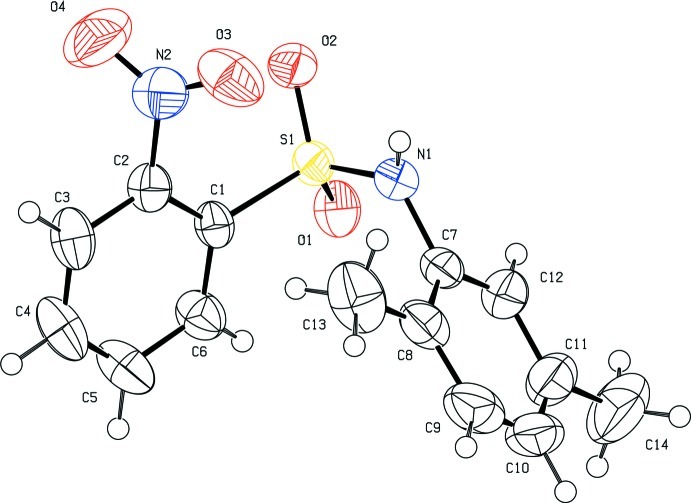
Molecular structure of the title compound, showing the atom labelling scheme and with displacement ellipsoids drawn at the 50% probability level.

**Fig. 2.**
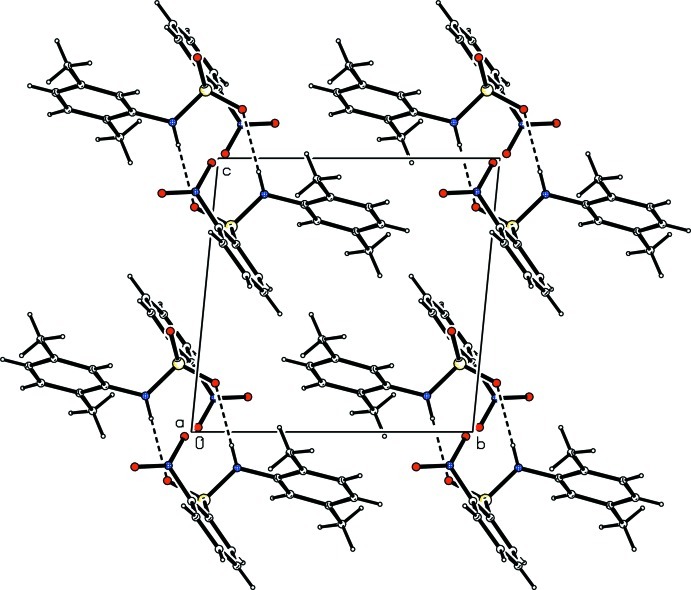
Molecular packing of the title compound with hydrogen bonding shown as dashed lines.

## supplementary crystallographic information

### Comment

As a part of studying the effect of substituents on the structures and other aspects of *N*-arylsulfonamides (Chaithanya *et al.*, 2012; Gowda *et al.*, 2002) and *N*-chloroarylsulfonamides (Gowda & Shetty, 2004; Shetty & Gowda, 2004), in the present work, the crystal structure of *N*-(2,5-dimethylphenyl)-2-nitrobenzene- sulfonamide (I) has been determined (Fig. 1). The conformation of the N—H bond is *syn* to the *ortho*-nitro group in the sulfonyl benzene ring and *anti* to the *ortho*-methyl and *syn* to the *meta*-methyl groups in the anilino ring, compared to the *anti* conformation observed between the N—H bond and both the *ortho*- and *meta*-methyl groups in the anilino ring observed in *N*-(2,3-dimethylphenyl)-2-nitrobenzene- sulfonamide (II) (Chaithanya *et al.*, 2012).

The molecules in (I) are twisted at the S—N bond with the torsional angle of 71.41 (18)°, compared to the values of -60.37 (30) and 58.81 (34)° in the two independent molecules of (II).

The dihedral angle between the sulfonyl and the anilino rings is 51.07 (8)°, compared to the values of 53.67 (8) and 56.99 (9)° in the two molecules of (II).

The amide H-atom showed the intermolecular H-bonding with the sulfonyl oxygen atom of the other molecule, generating inversion dimers (Table 1, Fig. 2.)

In the crystal structure, N1—H1N···O2(S) intermolecular hydrogen bonds link the molecules into inversion dimers (Table 1, Fig. 2.)

### Experimental

The title compound was prepared by treating 2-nitrobenzenesulfonyl- chloride with 2,5-dimethylaniline in the stoichiometric ratio and boiling the reaction mixture for 15 minutes. The reaction mixture was then cooled to room temperature and added to ice cold water (100 ml). The resultant solid *N*-(2,5-dimethylphenyl)-2-nitrobenzenesulfon- amide was filtered under suction and washed thoroughly with cold water and dilute HCl to remove the excess sulfonylchloride and aniline, respectively. It was then recrystallized to constant melting point from dilute ethanol. The purity of the compound was checked and characterized by its infrared spectra.

Prism like light brown single crystals of the title compound used in X-ray diffraction studies were grown in ethanolic solution by slow evaporation of the solvent at room temperature.

### Refinement

H atoms bonded to C were positioned with idealized geometry using a riding model with the aromatic C—H = 0.93 Å, methyl C—H = 0.96 Å. The amino H atoms were freely refined with the N—H distance restrained to 0.86 (2) Å. All H atoms were refined with isotropic displacement parameters set at 1.2 *U*_eq_(C-aromatic, N) and 1.5 *U*_eq_ (*C*-methyl) of the parent atom.

The (0 1 1) reflection is probably affected by the beamstop and was omitted from the refinement.

### Crystal data

**Table:**

C_14_H_14_N_2_O_4_S	*Z* = 2
*M**_r_* = 306.33	*F*(000) = 320
Triclinic, *P*1	*D*_x_ = 1.364 Mg m^−^^3^
Hall symbol: -P 1	Mo *K*α radiation, λ = 0.71073 Å
*a* = 8.1987 (7) Å	Cell parameters from 3120 reflections
*b* = 9.6729 (9) Å	θ = 2.6–27.8°
*c* = 9.9328 (9) Å	µ = 0.23 mm^−^^1^
α = 84.386 (9)°	*T* = 293 K
β = 72.096 (8)°	Prism, light brown
γ = 89.239 (9)°	0.36 × 0.24 × 0.16 mm
*V* = 745.86 (12) Å^3^	

### Data collection

**Table:**

Oxford Diffraction Xcaliburdiffractometer with Sapphire CCD	3027 independent reflections
Radiation source: fine-focus sealed tube	2629 reflections with *I* > 2σ(*I*)
Graphite monochromator	*R*_int_ = 0.011
Rotation method data acquisition using ω scans	θ_max_ = 26.4°, θ_min_ = 2.6°
Absorption correction: multi-scan (*CrysAlis RED*; Oxford Diffraction, 2009)	*h* = −10→10
*T*_min_ = 0.921, *T*_max_ = 0.964	*k* = −12→11
4954 measured reflections	*l* = −12→10

### Refinement

**Table:**

Refinement on *F*^2^	Primary atom site location: structure-invariant direct methods
Least-squares matrix: full	Secondary atom site location: difference Fourier map
*R*[*F*^2^ > 2σ(*F*^2^)] = 0.042	Hydrogen site location: inferred from neighbouring sites
*wR*(*F*^2^) = 0.108	H atoms treated by a mixture of independent and constrained refinement
*S* = 1.14	*w* = 1/[σ^2^(*F*_o_^2^) + (0.0379*P*)^2^ + 0.3685*P*] where *P* = (*F*_o_^2^ + 2*F*_c_^2^)/3
3027 reflections	(Δ/σ)_max_ < 0.001
193 parameters	Δρ_max_ = 0.28 e Å^−^^3^
1 restraint	Δρ_min_ = −0.29 e Å^−^^3^

### Special details

**Table:**

Experimental. Absorption correction: CrysAlis RED (Oxford Diffraction, 2009) Empirical absorption correction using spherical harmonics, implemented in SCALE3 ABSPACK scaling algorithm.
Geometry. All e.s.d.'s (except the e.s.d. in the dihedral angle between two l.s. planes) are estimated using the full covariance matrix. The cell e.s.d.'s are taken into account individually in the estimation of e.s.d.'s in distances, angles and torsion angles; correlations between e.s.d.'s in cell parameters are only used when they are defined by crystal symmetry. An approximate (isotropic) treatment of cell e.s.d.'s is used for estimating e.s.d.'s involving l.s. planes.
Refinement. Refinement of *F*^2^ against ALL reflections. The weighted *R*-factor *wR* and goodness of fit *S* are based on *F*^2^, conventional *R*-factors *R* are based on *F*, with *F* set to zero for negative *F*^2^. The threshold expression of *F*^2^ > σ(*F*^2^) is used only for calculating *R*-factors(gt) *etc*. and is not relevant to the choice of reflections for refinement. *R*-factors based on *F*^2^ are statistically about twice as large as those based on *F*, and *R*- factors based on ALL data will be even larger.

### Fractional atomic coordinates and isotropic or equivalent isotropic displacement parameters (Å^2^)

**Table:**

	*x*	*y*	*z*	*U*_iso_*/*U*_eq_	
C1	0.3540 (2)	0.0929 (2)	0.6857 (2)	0.0387 (4)	
C2	0.4684 (2)	0.0253 (2)	0.7467 (2)	0.0433 (4)	
C3	0.6437 (3)	0.0467 (3)	0.6890 (3)	0.0561 (6)	
H3	0.7187	−0.0016	0.7294	0.067*	
C4	0.7051 (3)	0.1400 (3)	0.5713 (3)	0.0657 (7)	
H4	0.8226	0.1564	0.5326	0.079*	
C5	0.5947 (3)	0.2092 (3)	0.5104 (3)	0.0657 (7)	
H5	0.6376	0.2732	0.4313	0.079*	
C6	0.4199 (3)	0.1846 (2)	0.5655 (2)	0.0525 (5)	
H6	0.3462	0.2299	0.5216	0.063*	
C7	0.0552 (3)	0.3225 (2)	0.8314 (2)	0.0448 (5)	
C8	0.1732 (3)	0.4106 (2)	0.8577 (2)	0.0567 (6)	
C9	0.1583 (4)	0.5520 (3)	0.8214 (3)	0.0760 (8)	
H9	0.2318	0.6147	0.8398	0.091*	
C10	0.0386 (4)	0.6010 (3)	0.7595 (3)	0.0772 (9)	
H10	0.0348	0.6961	0.7352	0.093*	
C11	−0.0768 (4)	0.5141 (3)	0.7317 (3)	0.0701 (7)	
C12	−0.0680 (3)	0.3728 (2)	0.7717 (2)	0.0546 (5)	
H12	−0.1464	0.3114	0.7580	0.065*	
C13	0.3075 (4)	0.3582 (3)	0.9231 (3)	0.0808 (9)	
H13A	0.3794	0.2942	0.8642	0.121*	
H13B	0.2531	0.3118	1.0159	0.121*	
H13C	0.3759	0.4350	0.9310	0.121*	
C14	−0.2091 (5)	0.5675 (4)	0.6626 (4)	0.1074 (12)	
H14A	−0.1524	0.6151	0.5709	0.161*	
H14B	−0.2833	0.6304	0.7213	0.161*	
H14C	−0.2756	0.4907	0.6518	0.161*	
N1	0.0568 (2)	0.17583 (17)	0.86856 (18)	0.0440 (4)	
H1N	0.054 (3)	0.147 (2)	0.9500 (18)	0.053*	
N2	0.4089 (3)	−0.0701 (2)	0.8773 (2)	0.0605 (5)	
O1	0.06849 (18)	0.11008 (16)	0.63232 (16)	0.0524 (4)	
O2	0.09053 (18)	−0.06778 (14)	0.82168 (17)	0.0530 (4)	
O3	0.3174 (2)	−0.0226 (2)	0.98338 (19)	0.0766 (6)	
O4	0.4595 (3)	−0.1884 (2)	0.8720 (3)	0.1039 (8)	
S1	0.12780 (6)	0.06923 (5)	0.75061 (5)	0.04028 (15)	

### Atomic displacement parameters (Å^2^)

**Table:**

	*U*^11^	*U*^22^	*U*^33^	*U*^12^	*U*^13^	*U*^23^
C1	0.0295 (9)	0.0461 (10)	0.0427 (10)	0.0008 (7)	−0.0130 (8)	−0.0090 (8)
C2	0.0392 (10)	0.0489 (11)	0.0462 (11)	0.0018 (8)	−0.0184 (8)	−0.0087 (9)
C3	0.0352 (10)	0.0803 (16)	0.0610 (14)	0.0081 (10)	−0.0235 (10)	−0.0184 (12)
C4	0.0340 (11)	0.107 (2)	0.0540 (13)	−0.0084 (12)	−0.0080 (10)	−0.0155 (13)
C5	0.0470 (12)	0.0948 (19)	0.0483 (13)	−0.0161 (12)	−0.0074 (10)	0.0054 (12)
C6	0.0427 (11)	0.0664 (14)	0.0482 (12)	−0.0028 (10)	−0.0159 (9)	0.0026 (10)
C7	0.0419 (10)	0.0408 (10)	0.0425 (10)	−0.0039 (8)	0.0003 (8)	−0.0034 (8)
C8	0.0508 (12)	0.0569 (13)	0.0516 (12)	−0.0120 (10)	0.0032 (10)	−0.0149 (10)
C9	0.0778 (18)	0.0586 (16)	0.0737 (18)	−0.0236 (14)	0.0073 (15)	−0.0178 (13)
C10	0.099 (2)	0.0401 (13)	0.0682 (17)	−0.0027 (14)	0.0094 (16)	−0.0016 (12)
C11	0.0859 (19)	0.0550 (15)	0.0579 (14)	0.0203 (13)	−0.0073 (13)	−0.0015 (11)
C12	0.0560 (13)	0.0474 (12)	0.0562 (13)	0.0057 (10)	−0.0110 (10)	−0.0070 (10)
C13	0.0585 (15)	0.101 (2)	0.090 (2)	−0.0091 (14)	−0.0240 (14)	−0.0389 (17)
C14	0.136 (3)	0.085 (2)	0.100 (3)	0.050 (2)	−0.041 (2)	0.0008 (19)
N1	0.0415 (9)	0.0426 (9)	0.0433 (9)	−0.0010 (7)	−0.0078 (7)	0.0007 (7)
N2	0.0507 (11)	0.0670 (13)	0.0716 (14)	−0.0038 (9)	−0.0350 (11)	0.0086 (10)
O1	0.0400 (7)	0.0664 (10)	0.0586 (9)	0.0051 (7)	−0.0254 (7)	−0.0112 (7)
O2	0.0451 (8)	0.0412 (8)	0.0708 (10)	−0.0064 (6)	−0.0158 (7)	−0.0025 (7)
O3	0.0671 (11)	0.1086 (16)	0.0516 (10)	−0.0154 (10)	−0.0196 (9)	0.0114 (10)
O4	0.1010 (17)	0.0670 (13)	0.147 (2)	0.0127 (12)	−0.0545 (16)	0.0241 (13)
S1	0.0304 (2)	0.0421 (3)	0.0500 (3)	−0.00165 (17)	−0.01472 (19)	−0.0047 (2)

### Geometric parameters (Å, º)

**Table:**

C1—C6	1.383 (3)	C9—H9	0.9300
C1—C2	1.389 (3)	C10—C11	1.380 (4)
C1—S1	1.7764 (18)	C10—H10	0.9300
C2—C3	1.384 (3)	C11—C12	1.392 (3)
C2—N2	1.470 (3)	C11—C14	1.512 (4)
C3—C4	1.371 (4)	C12—H12	0.9300
C3—H3	0.9300	C13—H13A	0.9600
C4—C5	1.369 (4)	C13—H13B	0.9600
C4—H4	0.9300	C13—H13C	0.9600
C5—C6	1.383 (3)	C14—H14A	0.9600
C5—H5	0.9300	C14—H14B	0.9600
C6—H6	0.9300	C14—H14C	0.9600
C7—C12	1.382 (3)	N1—S1	1.6049 (18)
C7—C8	1.397 (3)	N1—H1N	0.823 (16)
C7—N1	1.431 (3)	N2—O4	1.213 (3)
C8—C9	1.393 (4)	N2—O3	1.218 (3)
C8—C13	1.500 (4)	O1—S1	1.4242 (15)
C9—C10	1.367 (5)	O2—S1	1.4295 (15)
			
C6—C1—C2	118.12 (18)	C10—C11—C12	116.9 (3)
C6—C1—S1	117.31 (15)	C10—C11—C14	122.5 (3)
C2—C1—S1	124.57 (15)	C12—C11—C14	120.6 (3)
C3—C2—C1	121.6 (2)	C7—C12—C11	121.1 (2)
C3—C2—N2	116.78 (18)	C7—C12—H12	119.4
C1—C2—N2	121.63 (18)	C11—C12—H12	119.4
C4—C3—C2	119.0 (2)	C8—C13—H13A	109.5
C4—C3—H3	120.5	C8—C13—H13B	109.5
C2—C3—H3	120.5	H13A—C13—H13B	109.5
C5—C4—C3	120.4 (2)	C8—C13—H13C	109.5
C5—C4—H4	119.8	H13A—C13—H13C	109.5
C3—C4—H4	119.8	H13B—C13—H13C	109.5
C4—C5—C6	120.5 (2)	C11—C14—H14A	109.5
C4—C5—H5	119.7	C11—C14—H14B	109.5
C6—C5—H5	119.7	H14A—C14—H14B	109.5
C5—C6—C1	120.3 (2)	C11—C14—H14C	109.5
C5—C6—H6	119.9	H14A—C14—H14C	109.5
C1—C6—H6	119.9	H14B—C14—H14C	109.5
C12—C7—C8	121.8 (2)	C7—N1—S1	121.88 (14)
C12—C7—N1	117.78 (19)	C7—N1—H1N	119.0 (17)
C8—C7—N1	120.4 (2)	S1—N1—H1N	115.8 (17)
C9—C8—C7	116.1 (2)	O4—N2—O3	125.3 (2)
C9—C8—C13	121.2 (2)	O4—N2—C2	117.4 (2)
C7—C8—C13	122.6 (2)	O3—N2—C2	117.3 (2)
C10—C9—C8	121.9 (3)	O1—S1—O2	119.85 (9)
C10—C9—H9	119.1	O1—S1—N1	108.74 (9)
C8—C9—H9	119.1	O2—S1—N1	107.05 (9)
C9—C10—C11	122.1 (2)	O1—S1—C1	105.37 (9)
C9—C10—H10	118.9	O2—S1—C1	108.28 (9)
C11—C10—H10	118.9	N1—S1—C1	106.93 (9)
			
C6—C1—C2—C3	−0.9 (3)	C8—C7—C12—C11	1.7 (3)
S1—C1—C2—C3	178.74 (16)	N1—C7—C12—C11	−179.5 (2)
C6—C1—C2—N2	177.9 (2)	C10—C11—C12—C7	−2.4 (4)
S1—C1—C2—N2	−2.5 (3)	C14—C11—C12—C7	178.3 (2)
C1—C2—C3—C4	2.1 (3)	C12—C7—N1—S1	75.0 (2)
N2—C2—C3—C4	−176.8 (2)	C8—C7—N1—S1	−106.1 (2)
C2—C3—C4—C5	−1.2 (4)	C3—C2—N2—O4	−59.0 (3)
C3—C4—C5—C6	−0.8 (4)	C1—C2—N2—O4	122.2 (2)
C4—C5—C6—C1	2.0 (4)	C3—C2—N2—O3	118.6 (2)
C2—C1—C6—C5	−1.2 (3)	C1—C2—N2—O3	−60.2 (3)
S1—C1—C6—C5	179.19 (19)	C7—N1—S1—O1	−41.91 (18)
C12—C7—C8—C9	0.5 (3)	C7—N1—S1—O2	−172.73 (15)
N1—C7—C8—C9	−178.28 (19)	C7—N1—S1—C1	71.41 (18)
C12—C7—C8—C13	179.7 (2)	C6—C1—S1—O1	22.19 (19)
N1—C7—C8—C13	0.9 (3)	C2—C1—S1—O1	−157.44 (17)
C7—C8—C9—C10	−2.0 (4)	C6—C1—S1—O2	151.56 (17)
C13—C8—C9—C10	178.8 (2)	C2—C1—S1—O2	−28.1 (2)
C8—C9—C10—C11	1.3 (4)	C6—C1—S1—N1	−93.39 (18)
C9—C10—C11—C12	0.9 (4)	C2—C1—S1—N1	86.97 (18)
C9—C10—C11—C14	−179.7 (3)		

### Hydrogen-bond geometry (Å, º)

**Table:**

*D*—H···*A*	*D*—H	H···*A*	*D*···*A*	*D*—H···*A*
N1—H1*N*···O2^i^	0.82 (2)	2.27 (2)	3.023 (2)	152 (2)

Symmetry code: (i) −*x*, −*y*, −*z*+2.
